# Vaccination against GIP for the Treatment of Obesity

**DOI:** 10.1371/journal.pone.0003163

**Published:** 2008-09-09

**Authors:** Alma Fulurija, Thomas A. Lutz, Katja Sladko, Melania Osto, Peter Y. Wielinga, Martin F. Bachmann, Philippe Saudan

**Affiliations:** 1 Cytos Biotechnology AG, Schlieren, Switzerland; 2 Institute of Veterinary Physiology and Center of Integrative Human Physiology, Vetsuisse Faculty, University of Zurich, Zurich, Switzerland; University of Parma, Italy

## Abstract

**Background:**

According to the WHO, more than 1 billion people worldwide are overweight and at risk of developing chronic illnesses, including cardiovascular disease, type 2 diabetes, hypertension and stroke. Current therapies show limited efficacy and are often associated with unpleasant side-effect profiles, hence there is a medical need for new therapeutic interventions in the field of obesity. Gastric inhibitory peptide (GIP, also known as glucose-dependent insulinotropic polypeptide) has recently been postulated to link over-nutrition with obesity. In fact GIP receptor-deficient mice (GIPR^−/−^) were shown to be completely protected from diet-induced obesity. Thus, disrupting GIP signaling represents a promising novel therapeutic strategy for the treatment of obesity.

**Methodology/Principal Findings:**

In order to block GIP signaling we chose an active vaccination approach using GIP peptides covalently attached to virus-like particles (VLP-GIP). Vaccination of mice with VLP-GIP induced high titers of specific antibodies and efficiently reduced body weight gain in animals fed a high fat diet. The reduction in body weight gain could be attributed to reduced accumulation of fat. Moreover, increased weight loss was observed in obese mice vaccinated with VLP-GIP. Importantly, despite the incretin action of GIP, VLP-GIP-treated mice did not show signs of glucose intolerance.

**Conclusions/Significance:**

This study shows that vaccination against GIP was safe and effective. Thus active vaccination may represent a novel, long-lasting treatment for obesity. However further preclinical safety/toxicology studies will be required before the therapeutic concept can be addressed in humans.

## Introduction

Obesity has become one of the leading health problems worldwide. The global obesity epidemic results from a combination of genetic susceptibility, increased availability of high-energy foods and decreased requirement for physical activity in modern society [1]. Obesity and excess weight are major risk factors for chronic diseases, including type II diabetes, cardiovascular diseases, gastrointestinal disorders and certain forms of cancer. Importantly, body weight reduction in the range of 10% is associated with significant improvements in a wide range of co-morbid conditions [2]–[4]. Currently approved anti-obesity drugs show only limited efficacy, generally facilitating no more than a 5–10% reduction of body weight and are often associated with unpleasant side-effect profiles [5]–[7]. To date the only treatment leading to substantial, sustained body weight loss is bariatric surgery. However, this intervention is associated with between 1.5% and 4.5% mortality during the first three month following surgery [8]. Hence there is a major medical need for the development of new anti-obesity drugs. In the past decade our knowledge of gut hormones and their central role in the control of food intake and energy balance has substantially improved [9]–[11]. This increased understanding has led to the identification of new potential targets for pharmaceutical intervention.

Gastric inhibitory peptide, also known as glucose-dependent insulinotropic polypeptide (GIP) is one of these peptide hormones. GIP is a 42 amino acid, gastrointestinal polypeptide released from duodenal and jejunal K-cells after ingestion of nutrients and has been shown to facilitate the disposal of both glucose and fat [12]. GIP acts rapidly on pancreatic β-cells to stimulate the release of insulin thus ensuring prompt uptake of glucose into the tissue. In addition, GIP aids fat deposition and triglyceride accumulation in adipocytes. Specifically, GIP has been shown to promote triglyceride clearance from the circulation [13], [14], a process partly mediated by its ability to stimulate lipoprotein lipase activity [15]. Moreover, GIP receptors are expressed on adipocytes [16] consistent with a direct role of GIP on these cells. Recently, GIP receptor-deficient mice (GIPR^−/−^) were shown to be completely protected from diet-induced obesity [17]. Likewise, recent studies demonstrated that treatment with a GIP-receptor antagonist led to reduced weight gain in mice fed a high fat diet and weight loss in obese mice [18]–[20]. Hence, disrupting GIP signaling represents a promising, novel therapeutic strategy for the treatment of obesity.

The induction of GIP-specific, neutralizing antibodies through vaccination is a particularly attractive possibility, given that the blockade of GIP would be long-lasting. We have previously shown that antigens displayed on highly repetitive viral surfaces can break B cell tolerance [21] and epitopes displayed on the surface of virus-like particles (VLPs) are able to efficiently induce self-specific antibody responses in mice and humans [22]–[26]. In this study, we show that vaccination against GIP prevents excessive body weight gain in rodents fed a high fat diet and induces increased weight loss in obese mice. Hence, active vaccination may represent an attractive and convenient new therapy for the treatment of obesity.

## Results

### Vaccination against GIP results in high levels of GIP-specific antibodies

**Figure 1:**
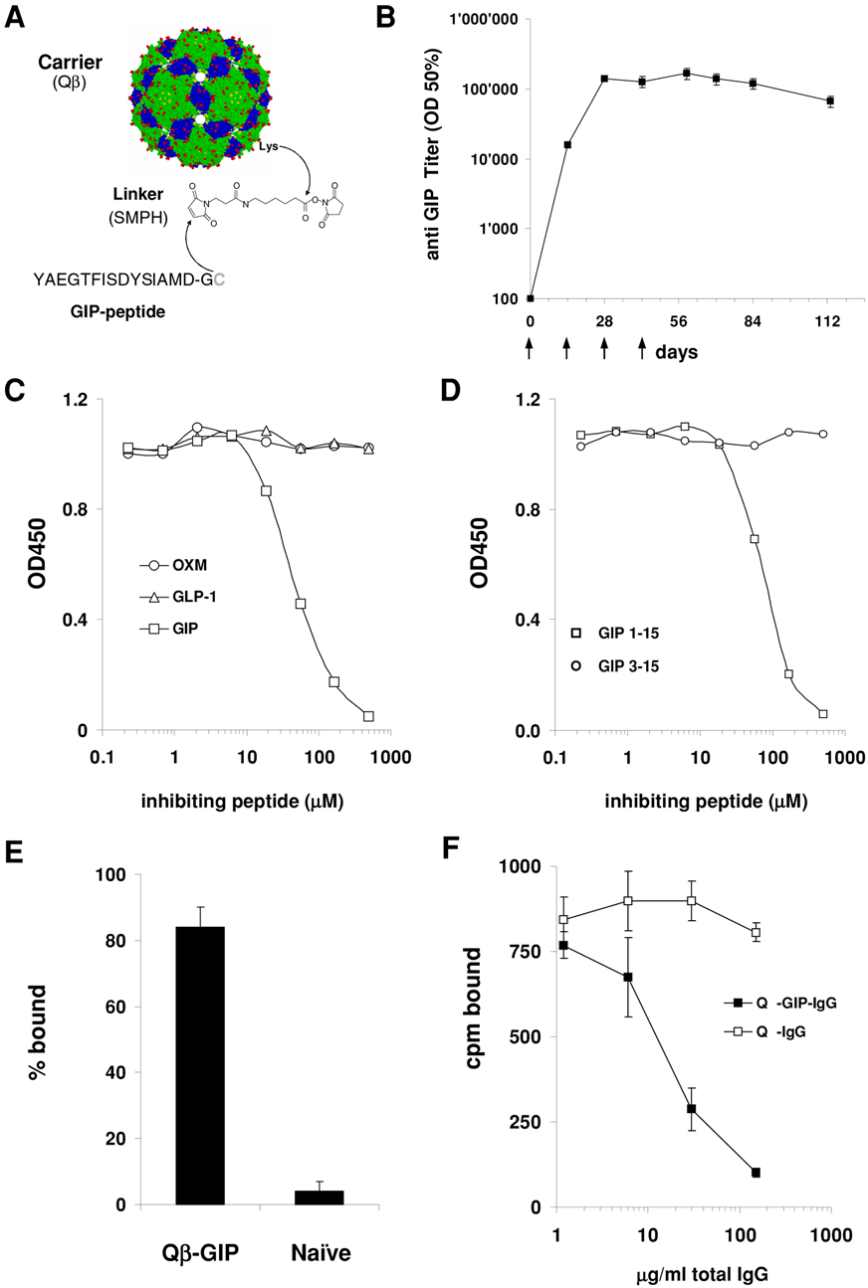
Vaccination against GIP results in high, specific antibody titers. (A) Schematic diagram of GIP peptide coupling to Qβ VLPs. GIP peptides (aa 1–15), corresponding to the N-terminus of the active peptide, were covalently linked to Qβ VLPs via an SMPH linker. (B) GIP-specific antibody titers in vaccinated mice. Female mice were immunized s.c. with 100 µg of Qβ-GIP (days 0, 14, 28 and 42). Average GIP-specific antibody titers±SEM (n = 6) at the indicated time points are shown. (C) Cross-reactivity of GIP-specific antibodies. A serum pool from Qβ-GIP-immunized mice was incubated with increasing concentrations of GIP, GLP-1 or oxyntomodulin (OXM). The amount of free antibody was quantified by ELISA. (D) Recognition of the N-terminus of full length GIP. A serum pool from Qβ-GIP-immunized mice was preincubated either with GIP1–15 or GIP3–15 and free antibodies quantified by ELISA. (E) Sequestration of GIP *in vivo*. Qβ-GIP immunized mice or naïve mice were challenged i.v. with 1 ng of I^125^-GIP. 30 minutes later the amount of antibody-bound GIP was determined. The percentage of antibody bound GIP±SEM (n = 4) is shown. (F) Antibody mediated blocking of GIP binding to its receptor. I^125^-GIP was incubated with purified total IgG from Qβ-GIP or Qβ immunized mice and added to CHOK1-GIPR cells and bound GIP determined after an overnight incubation at 4°C. The final concentration of GIP was 20 ng/ml and the concentration of total IgG is shown on the x-axis. Error bars represent standard deviations from triplicates.

To overcome GIP-specific B cell unresponsiveness, we covalently coupled peptides consisting of the first 15 amino acids of mature GIP to the highly repetitive surface of bacteriophage Qβ VLPs [24], [27] (Figure 1A). The resulting vaccine was named Qβ-GIP. Mice were immunized s.c. with 100 µg of Qβ-GIP, formulated in saline, on days 0, 14, 28 and 42. GIP-specific antibody titers were determined at regular intervals. After a single immunisation, high GIP-specific antibody titers were induced. Antibody levels further increased with subsequent injections and were maintained over a period of at least three months (Figure 1B). These results demonstrate that Qβ-GIP could overcome immunological tolerance resulting in the induction of high GIP-specific antibody titers. Since the N-terminal peptide used in Qβ-GIP shares approximately 50% homology with GLP-1 and oxyntomodulin, sera from vaccinated mice were analysed for cross-reactivity with these hormones in an inhibition ELISA. While pre-incubation with GIP efficiently prevented binding of GIP-specific antibodies to plate-coated GIP, neither GLP-1 nor oxyntomodulin inhibited binding of anti-GIP sera to GIP, demonstrating that the induced antibody response is highly specific (Figure 1C). Considering that the first two N-terminal amino acids of GIP are rapidly cleaved by dipeptidyl peptidase (DPP)-IV [28] *in vivo* we wanted to test whether the induced immune response by our vaccine also recognizes the N-terminally cleaved form of GIP which has been shown to be a partial antagonist of GIP *in vitro* [29]. To test this, a competition ELISA with GIP coated plates was performed with sera from Qβ-GIP immunised mice pre-incubated either with the peptide used for immunisation (GIP1–15) or an N-terminally truncated version of the peptide (GIP3–15). As shown in Figure 1D, GIP1–15 efficiently competed serum binding to plate coated GIP, whereas GIP3–15 failed to do so even at very high concentrations. These results demonstrate that with the vaccine used here, most of the immune response is directed towards an intact N-terminus of GIP.

Measurement of GIP concentration in serum revealed that Qβ-GIP immunized mice displayed significantly higher GIP levels than Qβ immunized control mice (data not shown) most likely due to stabilization of GIP by the induced antibody response. Hence we set out to test whether the induced antibodies could still neutralise physiological concentrations of GIP *in vivo*. To this end Qβ-GIP immunised animals and control animals were challenged i.v. with 1 ng of I^125^-GIP and 30 minutes after the injection animals were culled, serum collected and antibody bound and free I^125^-GIP determined. As shown in Figure 1E, in vaccinated mice roughly 85% of the radiolabeled GIP was antibody bound whereas only 4% were found associated with the antibody fraction in naïve mice. This result demonstrates that the induced antibody response can efficiently sequester GIP *in vivo*. In similar experiments performed with I^125^-GLP-1 or I^125^-Oxyntomodulin only background levels of the radiolabelled ligands were found associated with the antibodies, further demonstrating that the induced antibody response is highly specific for GIP (Figure S1). Having demonstrated that the induced antibodies efficiently bound GIP in the serum we tested whether specific antibodies would prevent the interaction of GIP with its receptor. Hence, CHOK1 cells expressing the human GIP receptor were generated and used for *in vitro* receptor binding studies. Increasing amounts of purified IgGs from Qβ-GIP or Qβ immunised mice were incubated with a fixed amount of I^125^-GIP and added to receptor expressing cells. After overnight incubation at 4°C receptor bound GIP was determined. As shown in Figure 1F the induced GIP specific antibodies efficiently prevented GIP binding to its receptor. In contrast, purified IgG from Qβ immunized mice did not influence GIP receptor binding. Only very low levels of unspecific binding (∼50 cpm) of GIP to the parental CHOK1 cells were observed in the presence or absence of purified IgG from Qβ-GIP or Qβ immunised mice (data not shown). Taken together these results show that vaccination with Qβ-GIP induces highly specific antibodies which can sequester GIP and prevent its binding to the GIP receptor.

### Vaccination against GIP protects against diet-induced obesity

**Figure 2:**
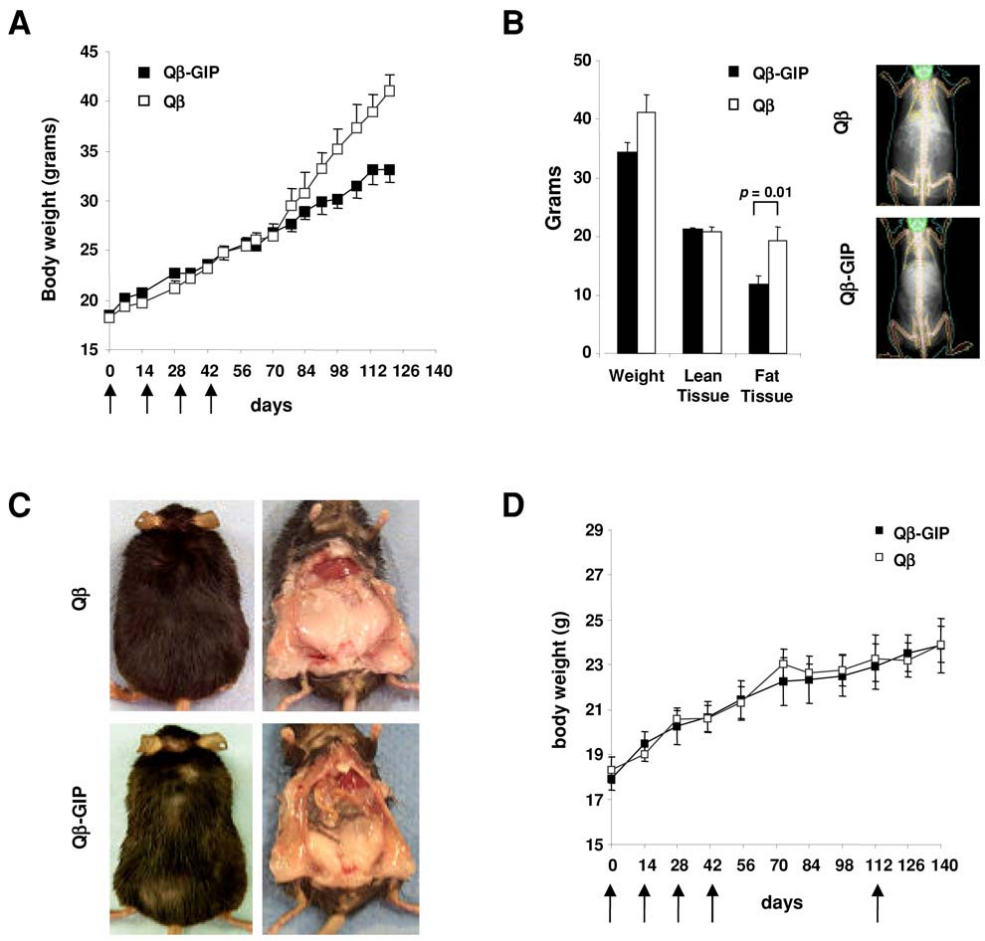
Vaccination against GIP protects against diet-induced obesity. (A) Body weight gain in immunized mice. Female mice were immunized (days 0, 14, 28, 42 and 133) with 100 µg of Qβ-GIP or Qβ VLPs and placed on a high fat diet (35% fat w/v). The average body weight+/−SEM (n = 6) is shown. Body weight gain was was significanty reduced in Qβ-GIP- compared to Qβ VLP-immunized animals from day 70 onwards (two way ANOVA F(1,80) = 18.55, p<0.0001). (B) Body composition of mice shown in (A) was measured by DEXAscan on day 142. Average total body mass, lean and fat tissue mass+/−SEM (n = 6) are shown. A significant reduction in fat content (*p* = 0.01) was observed between the Qβ-GIP- and Qβ-vaccinated group as determined by t-test. DEXAscan images of one representative animal per group are shown. (C) Macroscopic analysis of mice after 142 days of treatment with Qβ-GIP or Qβ VLPs. A representative mouse from each group from the experiment described in Figure 3A–B is shown. (D) Body weight gain in immunized mice on a standard rodent diet. Female mice were immunized (days 0, 14, 28, 42 and 112) with Qβ-GIP or Qβ VLPs and fed a standard diet (4% fat w/v) during the whole experiment. Average body weights+/−SEM (n = 5) are shown. No significant difference between the two experimental groups was observed as determined (two way ANOVA F(1/88) = 0.81, p = 0.6751).

Having established that Qβ-GIP induces a strong antibody response which can efficiently sequester GIP we wanted to investigate whether vaccination against GIP could reduce body weight gain in rodents. Adult, female mice were immunized with Qβ-GIP or control Qβ VLPs and placed on a high fat diet (35% fat w/v). As shown in Figure 2A, Qβ-GIP-vaccinated animals displayed significantly reduced body weight gain compared to Qβ-vaccinated animals. In fact, 4 months after the first vaccination, Qβ-GIP-treated mice had gained 8 g less weight than control animals. This corresponds to a 35% reduction in weight gain from start of the experiment. Next, body composition in these animals was analyzed by dual energy X-ray absorption scan (DEXAscan) on day 142. Whereas Qβ-vaccinated control mice displayed a fat content of 47%, the fat content of Qβ-GIP vaccinated mice was 34%. Hence, the body fat content in Qβ-GIP vaccinated mice was reduced by 28% (Figure 2B). In contrast, lean body mass was unaffected. Consequently, the reduction in body weight gain observed in Qβ-GIP-vaccinated mice was exclusively due to decreased fat accumulation. The difference in body weight gain and fat accumulation was also macroscopically very evident (Figure 2C). Since GIP is a self molecule produced by K-cells, we wanted to rule out an auto-inflammatory reaction in the gut as cause for the reduced body weight gain observed in these animals. Histological evaluation of gut sections revealed no evidence of inflammation (Figure S2). We further tested the effect of vaccination against GIP on age-related body weight gain in females fed a standard rodent diet (4% fat w/v). Both Qβ-GIP- and Qβ-vaccinated mice showed a similar age-related increase in body weight (Figure 2D). Hence, vaccination against GIP specifically prevents excessive body weight gain in rodents fed a high fat diet.

### Vaccination against GIP increases energy expenditure

**Figure 3:**
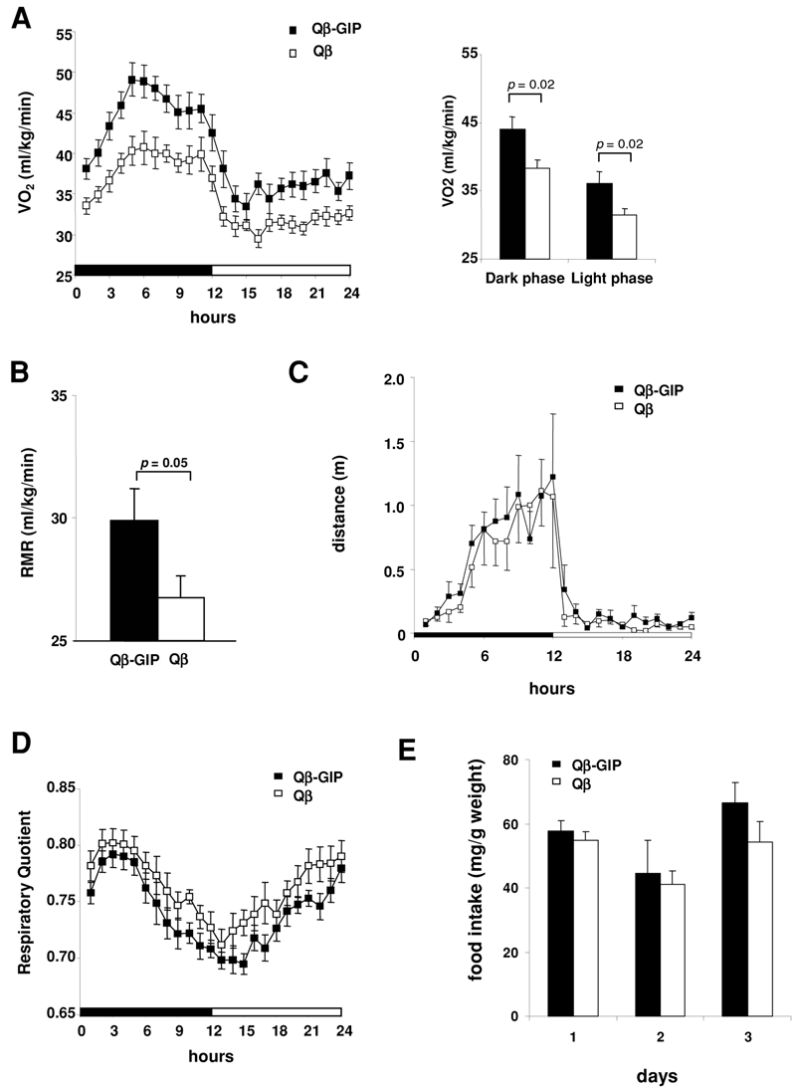
Vaccination against GIP increases energy expenditure and metabolic rate. Indirect calorimetry in immunized mice. Female mice were immunized (days 0, 14, 28, 42 and 125) with Qβ-GIP (n = 8) or Qβ VLPs (n = 10) and placed on a high fat diet. Indirect calorimetry was performed on half of the group on day 128 and on the other half on day 139. Combined data from these measurements are shown. (A) Oxygen consumption (VO2). The left panel shows average oxygen consumption+/−SEM. Qβ-GIP-vaccinated animals display statistically, significantly increased VO2 (p<0.0001) over the 24 h period. Average oxygen consumption+/−SEM during the dark and light phase is shown on the right. VO2 was significantly increased in Qβ-GIP-vaccinated animals compared to Qβ controls in both the dark (p = 0.02) and light phase (p = 0.02). (B) Resting metabolic rate (RMR). RMR was increased in Qβ-GIP-vaccinated animals compared to Qβ VLP controls (p = 0.05). (C) Physical activity was determined by measuring beam brakes over a 24 h period. No significant differences were observed between the two experimental groups. (D) Respiratory quotient (RQ) was measured for 24 hours during the dark and light phase. Average RQ±SEM is shown. RQ is defined as VCO2 (L)/VO2 (L). The difference observed between the two experimental groups did not reach statistical significance. (E) Food intake. Food intake was monitored over three consecutive day after the energy expenditure experiment. Average daily food intake in mg/g body weight+/−SEM (n = 5 are shown). No statistically significant difference was observed between the experimental groups (p>0.05). All statistical analyses were performed by two-sided t-tests.

To further elucidate why animals vaccinated against GIP gained less body weight, food intake, physical activity and energy expenditure were measured after 4 months on a high fat diet. Qβ-GIP-vaccinated mice showed significantly higher energy expenditure compared to control mice in both the dark and the light phase (Figure 3A). This can best be explained by an increase in basal metabolism, since resting metabolic rate was significantly higher in Qβ-GIP-vaccinated animals (Figure 3B) and no significant increase in physical activity was observed (Figure 3C). Moreover, Qβ-GIP-vaccinated animals displayed a lower respiratory quotient (RQ) throughout the experimental period, indicative of preferential burning of fat in the treated group. However, the observed difference in RQ did not reach statistical significance (Figure 3D). No differences in food intake were observed between the experimental groups determined during three consecutive days after the energy expenditure experiment (Figure 3E). Taken together these data indicate that the reduced body weight gain in Qβ-GIP-vaccinated mice fed a high fat diet was rather due to higher energy expenditure than lower energy intake or increased activity.

### Glucose homeostasis is unaffected after vaccination against GIP

**Figure 4:**
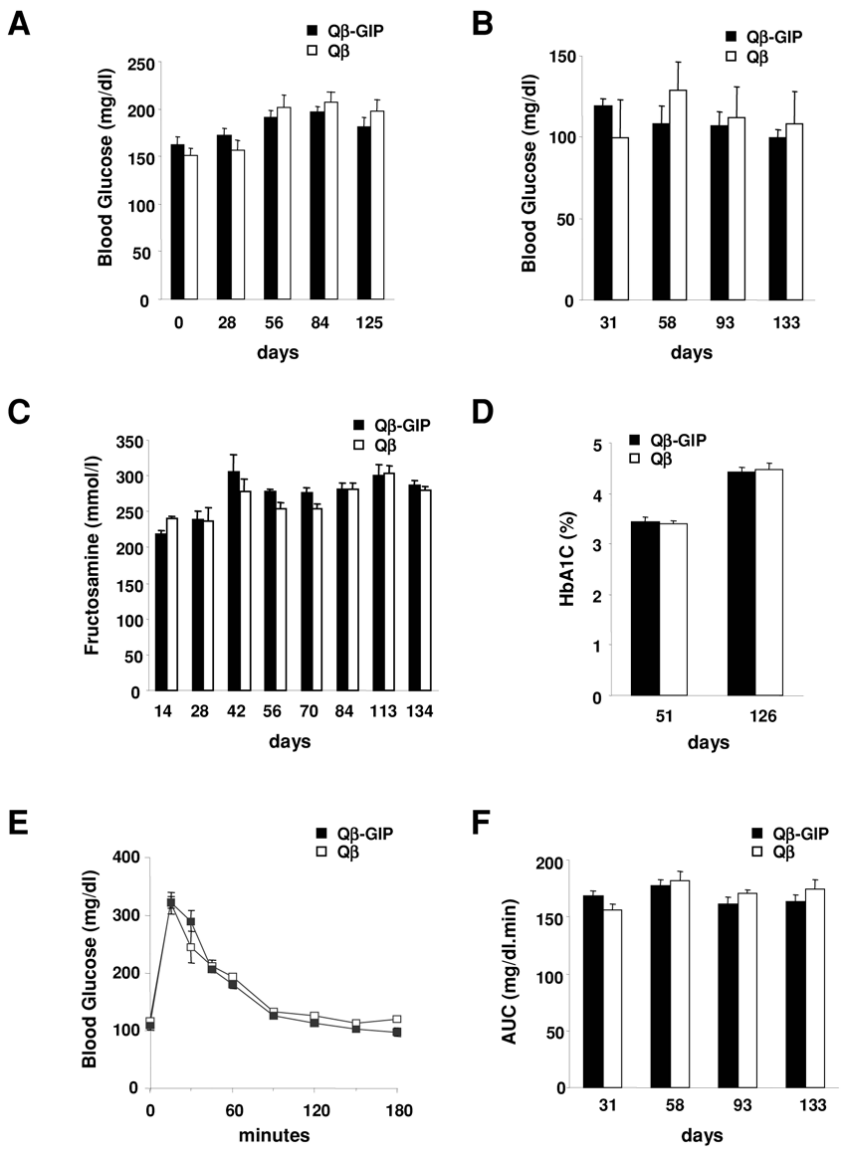
Vaccination against GIP does not alter glucose homeostasis. (A) Non-fasted blood glucose levels. Female mice were immunized (days 0, 14, 28, 42 and 133) with Qβ-GIP or Qβ VLPs and placed on a high fat diet and non-fasted glucose levels were measured at the indicated time points. Average blood glucose level per group±SEM (n = 10) are shown. No significant difference between vaccinated and control group was observed (two way ANOVA F(1, 72) = 0.06, p = 0.8094) (B) Fasted blood glucose levels. Female mice were immunized (days 0, 14, 28, 42) with Qβ-GIP or Qβ VLPs and placed on a high fat diet. After a 16 h fast, blood glucose levels were measured in the afternoon. Average blood glucose level per group±SEM (n = 5) are shown. No significant difference between vaccinated and control group was (two way ANOVA F(1, 24) = 0.21, p = 0.6553) (C) Average fructosamine concentrations±SEM (n = 6) from mice in Figure 2A are shown. No significant difference between the two experimental groups was observed (two way ANOVA F(1,70) = 1.49, p = 0.25) (D) Average HbA1c concentrations±SEM (n = 10) in mice immunized (days 0, 14, 28, 42) with Qβ-GIP or Qβ VLPs and fed a high fat diet are shown. No significant difference between vaccinated and control group was observed as determined by two-sided t-test. (E–F) Female mice were immunized (days 0, 14, 28, 42 and 122) with 100 µg of Qβ-GIP or Qβ VLPs and placed on a high fat diet. (E) Blood glucose levels during OGTT on day 142. Average blood glucose levels±SEM (n = 5) at the indicated time points are shown. No significant differences were observed between vaccinated and control animals as determined by two-sided t-test (F) Area under the curve during OGTT on days 31, 58, 93 and 133. Blood glucose levels were measured during OGTT and area under the curve calculated for individual animals. Average AUC±SEM (n = 5) for each group is shown. No significant difference was observed between the vaccinated and control group as determined by two-sided t-test.

Since GIP is a key incretin, it was necessary to test whether vaccination against GIP may disturb glucose homeostasis. First, non-fasted and fasted blood glucose levels were measured at regular intervals in vaccinated mice on a high fat diet. No significant differences in blood glucose levels were observed between Qβ-GIP- and Qβ-vaccinated animals (Figure 4A–B), suggesting that vaccination against GIP does not interfere with glucose homeostasis. To further explore overall blood glucose levels, serum samples from vaccinated mice were collected at bi-weekly intervals and the fructosamine content determined. Fructosamine levels provide a retrospective reading of blood glucose for the 2 weeks prior to measurement [30], [31]. As shown in Figure 4C, no significant difference between the Qβ-GIP- and Qβ-vaccinated mice was seen. Similar observations were made for HbA1c levels, which provide a retrospective picture of average blood glucose levels up to 3 months before analysis (Figure 4D). Taken together, these results demonstrate that active vaccination against GIP does not result in increased blood glucose levels. To further elucidate the consequences of ablation of GIP on glucose homeostasis, vaccinated mice fed a high fat diet were subjected to oral glucose tolerance tests (OGTT). OGTT were performed at the end of the experiment on day 142 in Qβ-GIP- and Qβ-immunized mice. Glucose was eliminated in both experimental groups with similar kinetics, indicating that glucose tolerance was not impaired by vaccination against GIP (Figure 4E). Additionally, OGTT were performed at monthly intervals. As shown in Figure 4F, no difference in oral glucose tolerance was observed between Qβ-GIP- and Qβ VLP-immunized mice throughout the experiment. Taken together, these findings suggest that active vaccination against GIP prevents excessive body weight gain and adiposity without altering glucose homeostasis.

### Vaccination against GIP does not interfere with lipid metabolism

**Figure 5:**
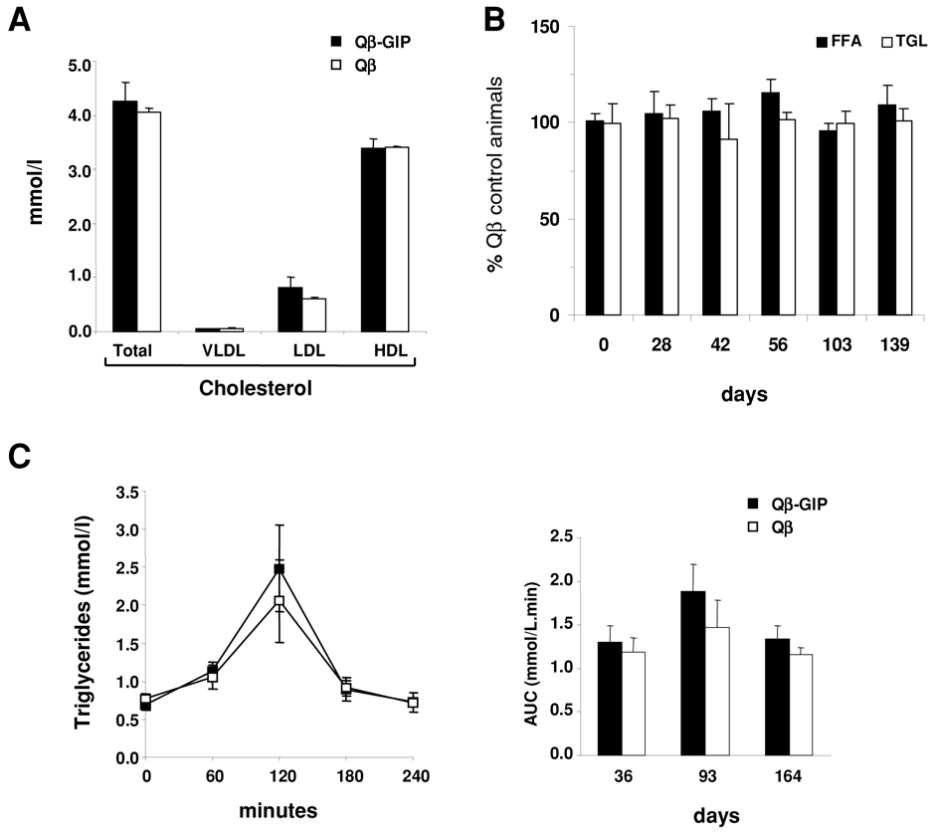
Vaccination against GIP does not alter lipid metabolism. (A–C) Female mice were immunized (days 0, 14, 28, 42) with Qβ-GIP or Qβ VLPs and placed on a high fat diet. (A) Serum lipid profile in mice on day 122. Total, vLDL, LDL and HDL cholesterol was measured. Average values±SEM (n = 10) are shown. No significant differences were observed between the groups (p>0.05). (B) Serum TGL and FFA profiles in mice. The percentage of control animals immunized with Qβ VLPs±SEM (n = 5) at the indicated time point are shown for triglycerides and free fatty acids. No significant differences in TGL or FFA levels were observed between the groups (p>0.05). (C) Postprandial lipid clearance in mice. Female mice were immunized (days 0, 14, 28, 41 and 126) with Qβ-GIP or Qβ VLPs and placed on a high fat diet. OLTT were performed at the indicated time points. The left panel shows the OLTT performed on day 36. AUC for all investigated time points is shown on the right. Average triglyceride levels or AUC±SEM (n = 5) are shown for each group. No significant differences were observed between the groups (p>0.05). All statistical analyses were performed by two sided t-tests.

To elucidate the effects of vaccination against GIP on lipid metabolism, serum lipid profiles were determined in vaccinated animals fed a high fat diet 4 months after the first injection. No significant differences were observed in total cholesterol, HDL, LDL or vLDL in Qβ-GIP-immunized mice compared to control mice (Figure 5A). Likewise, triglyceride and free fatty acid profiles were monitored at regular interval in vaccinated and control animals placed on a high fat diet. As shown in Figure 5B, no significant difference between the two experimental groups was observed. Since GIP is known to promote triglyceride clearance from the circulation [13], [14], we examined whether postprandial lipid clearance was affected in vaccinated animals. Oral lipid tolerance tests (OLTT) were performed in vaccinated mice fed a high fat diet on days 36, 93 and 163. Olive oil was administered by oral gavage and the TGL concentration in the blood determined at the indicated time points. Lipids were eliminated with similar kinetics in both experimental groups, indicating that TGL elimination was not impaired by vaccination against GIP (Figure 5C)

## Discussion

Here we describe a potential new treatment for obesity based on immunoneutralization of GIP, a gut hormone that has recently been shown to link over-nutrition to obesity [17]. GIP is a particularly attractive target since it is a peripheral hormone released into the circulation, where it can be easily captured by antibodies. Our approach is based on active vaccination using VLPs displaying GIP peptides on their surface. The highly repetitive display of peptides together with the strong T-helper cell epitopes provided by the VLP allowed for self tolerance to be overcome and led to a potent antibody response against GIP. Detailed analysis revealed that the induced antibodies were highly specific, since they showed no cross-reactivity with the closely related peptide hormones, GLP-1 and oxyntomodulin. As anticipated from studies in GIPR^−/−^ mice, Qβ-GIP vaccination resulted in reduced body weight gain and reduced fat accumulation in mice fed a high fat diet. In contrast, active vaccination against GIP did not affect normal age-related body weight gain in mice fed a standard rodent diet. These findings are in accordance with previous findings in GIPR^−/−^ mice showing little [17] or no difference [32] in age-related body weight gain in mice fed normal chow. Interestingly in the study shown here, the weight of Qβ-GIP immunized mice started to diverge from the control mice, only roughly 6 weeks after high titers had been reached around 70 days after the first immunization. This apparent delayed response is best explained by the observation that mice fed normal chow display a similar weight gain as mice fed a high fat diet during the first 10 weeks of the experiment (week 8–18). Only after 10–12 weeks animals fed a high fat diet start to significantly gain more weight and become obese. Similarly, Miyawaki and colleagues observed divergence of animals fed a high fat diet compared to animals fed a normal chow after only relatively late after 10–12 weeks of diet at an age of 18 weeks [17]. Hence, these observations suggest that only after prolonged high fat feeding excess fat in the diet is stored in the form of adipose tissue. Taken together these observations suggest that GIP maximizes the accumulation of fat tissue when energy rich food is consumed. This is of particular interest, since one of the major reasons for the increased incidence of obesity in humans is the unlimited access and consumption of energy rich food. The reduced body weight gain is best explained by the observed increase in energy expenditure, which was independent of physical activity, since no changes were observed between treated and control animals for this parameter. Although, at the end of the experiment no differences in food intake were observed, we cannot rule out a difference in food intake between the experimental groups early in the experiment which might account for parts of the differences in weight gain observed. In contrast to our findings, Hansotia *et al.* recently reported increased energy expenditure in single and double incretin receptor knockout mice, which could partly be attributed to increased locomotor activity in the dark phase [32]. Like the present study, they also observed an increase in resting metabolic rate in GIPR^−/−^ mice. Thus, GIP appears to affect energy expenditure by reducing resting metabolic rate and activity. The fact that we saw a trend with no statistically significant effect on locomotor activity, but found significant differences in resting metabolic rate, might be explained by different GIP thresholds needed to exert these effects. In the case of active vaccination against GIP, where GIP is unlikely to be completely blocked, resting metabolic rate is mostly affected.

While beneficial in reducing body weight gain, vaccination against GIP, a self-antigen, may raise concerns associated with the induction of auto-immune reactions. The risk for undesired auto-inflammatory responses is minimized with our vaccine [33]. First, a soluble molecule is targeted minimizing antibody-dependent cytotoxicity. Second, T-helper cell epitopes are provided by the carrier and a short GIP target peptide strongly reduces the likelihood of auto-reactive T-cells being induced by the vaccine. Nevertheless, detailed histopathology was performed in vaccinated animals and no signs of gut-specific inflammation or vaccine-related damage in other visceral organs was observed.

Since GIP is a major incretin, another safety concern is a disturbance of glucose homeostasis. In fact, GIPR^−/−^ mice have been reported to display signs of glucose intolerance [34]. Likewise, similar observations were made in lean mice after prolonged treatment with a GIP antagonist [35]. Hence, particular attention was paid to blood glucose homeostasis by measuring basal blood glucose, fructosamine, HbA1c and oral glucose tolerance in vaccinated and control animals. None of the parameters were negatively affected by vaccination against GIP. These findings are in contrast to observations made in GIPR^−/−^ mice and with the GIP antagonist mentioned above. It is also important to note that previous observations in GIPR^−/−^ mice and with GIP antagonists were made in lean animals. Recently, Gault *et al.* reported that prolonged treatment of mice fed a high fat diet with a GIP antagonist improved glucose tolerance in these animals suggesting that reducing GIP signaling could be beneficial under these circumstances [18]. In this reports Glucose tolerance was assessed after intraperitoneal glucose challenge which is not dependent upon GIP signaling and hence the improvement on glucose levels can be rather attributed to improved insulin signaling in this animals. Moreover McClean and colleagues investigated GIP antagonism in mice that had been fed a high fat diet for 160 days prior to the treatment with a GIP antagonist. Interestingly in these mice they found that a 50 day treatment with (Pro^3^)GIP led to weight loss, and significant improvement of glucose tolerance after both, i.p. challenge and feeding, suggesting that in severely obese mice antagonism of GIP is even beneficial for glucose homeostasis [20]. Hence, removal of GIP signaling appears to affect glucose homeostasis differently depending on the nutritional state of the animals. Whereas GIPR−/− animals show glucose intolerance on normal chow, the more recent work of McClean and colleagues show improvement of these parameters with their antagonists in severely obese mice after prolonged high fat feeding. Here these parameters were investigated in mice fed a high fat diet and most of our measurement were performed in between those two extreme situations. Hence, in view of these observations, the lack of notable effect on oral glucose tolerance in our experiments may not be that unexpected. Alternatively the differences observed here may be due to an incomplete neutralization of GIP by induced antibodies, still allowing for partial signaling to occur.

Investigations of lipid metabolism revealed no changes in vLDL-, LDL- and HDL-cholesterol concentrations in the serum of Qβ-GIP-vaccinated mice. Likewise, triglyceride and free fatty acid levels, as well as postprandial lipid clearance were not changed in vaccinated animals. Taken together, this study shows that active immunization against GIP leads to a strong reduction in body weight gain in mice on a high fat diet and without deteriorating blood glucose or lipid homeostasis.

Moreover in a preliminary experiment performed in obese male mice, suggests that active vaccination against GIP not only prevents excessive weight gain in animals fed a high fat diet but can also enhance weight loss in obese mice (Figure S3).

The role of GIP in glucose and lipid metabolism is well documented in several animal species [12], [36]. Likewise, the incretin activity of GIP and GLP1 is well established in humans [37]–[39]. Based on these observations DPP-IV inhibitors have been developed and drugs like Sitagliptin and Vildagliptin are now on the market as a novel class of type II diabetes drugs [40]–[42]. These antagonist, prevent the specific cleavage of the incretin hormones thereby increasing their half-lives and leading to increased insulin secretion. Interestingly, GIP has been reported to have a reduced incretin effect in type II diabetic patients whereas the insulinotropic effect of GLP is preserved in this patient population [43]–[45]. These findings suggest on one hand that the major effect of DPP-IV antagonist is mediated by the stabilization of GLP1 and on the other hand that elimination of GIP should not have a major impact on glucose homeostasis in type II diabetic patients. However, whereas the incretin effect of GIP has been described in detail in humans further investigations will be required to understand the potential role of GIP in linking over-nutrition with obesity in man. Hence, provided that further studies demonstrate a role of GIP in linking over nutrition to the development of obesity in humans, and further preclinical safety studies do not reveal toxic effects of concern, we believe that active vaccination against GIP may have the potential to be a convenient therapy for the treatment of obesity.

## Materials and Methods

### Animals

8 week old, C57BL/6 mice (∼20 g) were purchased from Harlan Netherlands (Horst, The Netherlands). Animals were housed in a pathogen free facility and were allowed to acclimatize for 2 weeks prior to immunization. In all experiments mice were caged in groups with the exception of the period during which energy expenditure and food intake was determined. The therapeutic experiment shown in Figure S3 was performed with male mice, all other experiments were performed with female mice. Unless otherwise indicated animals were fed a high fat diet (35% fat w/v (Diet 2127), Provimi Kliba, Switzerland), *ad libitum*, for the duration of the experimental period and had free access to water. Experiments were in accordance with Swiss Federal Veterinary Office (BVET) guidelines.

### Vaccine production

GIP fragment 1–15 including a GC linker sequence fused to the C-terminus of the GIP fragment (YAEGTFISDYSIAMDGC) was chemically synthesized according to standard procedures and coupled to Qβ VLPs as previously described [46], [47]. Briefly, A solution of 1 ml of 2.0 mg/ml Qβ VLPs in 20 mM Hepes, 150 mM NaCl pH 7.2 was reacted for 30 minutes with 67.2 µl of a 50 mM stock solution of SMPH (Pierce) in DMSO at 25°C. The reaction solution was subsequently dialyzed twice for 2 hours against 2 L of 20 mM Hepes, 150 mM NaCl, pH 7.2 at 4°C. Then 1 ml derivatized Qβ VLP was reacted with 286 µl of a 10 mM peptide solution for 2 hours at 20°C in 20 mM Hepes, 150 mM NaCl, pH 7.2. The coupling reactions were then centrifuged at 13 000 rpm for 5 minutes and the supernatants were collected and dialyzed once for 2 hours and then overnight against 2 L of 20 mM Hepes, 150 mM NaCl, pH 7.2 at 4°C. The covalent chemical coupling of GIP peptides to Qβ VLPs was assessed by SDS-PAGE using 12% Nu-PAGE gels (Invitrogen). Coomassie blue stained gels of the coupling reaction demonstrated the appearance of bands with molecular weights corresponding to those predicted for GIP peptides covalently linked to QβVLPs Coupling bands corresponding to one, two, three or four peptides coupled per subunit could be identified. Coupling efficiency [i.e. mol Qβ-GIP/mol Qβ monomer (total)] was estimated, by densitometric analysis of the Coomassie blue stained SDS-PAGE, to be between 1.6–2.2 GIP fragments per Qβ monomer.

### Immunization and in vitro *assays*

Animals were immunized subcutaneously with 100 µg (mice) or 300 µg (rats) of Qβ-GIP or Qβ VLPs, diluted in 200 µl PBS, at the indicated time points. GIP-specific antibody titers were determined by ELISA according to standard protocols using porcine GIP (Bachem #H-6220) at a concentration of 2.5 µg/ml for coating. The ELISA titer is defined as the reciprocals of the serum dilution needed to reach half the maximum optical density at saturation. For competition ELISAs, a serum pool from Qβ-GIP-immunized mice was diluted 1∶2500 and incubated with increasing concentrations of porcine GIP (Bachem #H-6220), murine GLP-1 (Bachem #H6795) or murine oxyntomodulin (Bachem #H6058). After overnight incubation, the amount of free antibody was quantified in a GIP-specific ELISA. For *in vivo* binding experiments, Qβ-GIP-immunized and naïve mice were challenged intravenously with 1 ng I^125^-GIP (Bachem H-5016) and 30 minutes after challenge animals were sacrificed and serum collected by cardiac puncture. Total IgG was pulled down from serum samples with protein G beads (Amersham, #17-0618-02). Protein G beads associated and free radioactivity was measured and the percentage antibody bound GIP calculated.

#### Generation of CHOK1 cells over-expressing the GIP receptor and binding studies

The human GIP receptor was amplified by RTPCR from a 3T3 cell line expressing the human GIP receptor (a kind gift from Dr. Jens Holst) using primer ATTTAATTAAGGCGCGCCACCATG ACTA CCTCTCCGATCC as forward and AATTAATTAACTCGAGCT AGCAGTAACTTTCCAAC TCC as reverse primer. The PCR product was then cloned into pBP [48]. The resulting construct was named pBP-GIPR. VSV G pseudotyped retroviruses were made by co-transfection of pBP-GIPR with pVPack-GP and pVPack-VSV-G (Stratagene) according to manufacturer's instruction. 48 h after transfection supernatants were collected and added to CHOK1 cells. One day later cells were passaged under puromycin selection (10 µg/ml). Transduced cell populations were than cultivated under selective pressure. For binding assays, 1×10^5^ CHOK1-GIPR/well were seeded 2 days prior to binding assays in 24 well plates in each well. Blocking assays were performed in a final volume of 500 µl. I^125^-GIP (20 ng/ml) was incubated in the presence of the indicated amounts of purified total IgG form Qβ-GIP or Qβ immunized mice in binding buffer (DMEM, 1% BSA, 10 mM Hepes ph 7.4) for 1 h at 4°C. This solution was than added to adherent cells and the cells were incubated over night at 4°C. Cells were then washed with binding buffer, resuspended in 500 µl 0.1 M NaOH and transferred to 1.5 ml scintillation fluid and bound GIP determined with a β-counter.

### Body weight and body composition analysis

Animals were weighed using a high precision scale. Body composition was determined by dual energy X-ray absorption scan (Piximus Series Densitometer, GE Medical Systems, Madison, USA) at the ICS (Illkirch, France).

### Glucose and lipid homeostasis

Total blood glucose, cholesterol, TGL, HDL, LDL, vLDL and FFA levels were measured after a 16 h fast. BGL were determined using a glucometer (Accu-Chek Aviva, Roche). Cholesterol, TGL and FFA levels were determined by enzymatic assays on an Olympus AU400 automated laboratory work station at the ICS (Illkirch, France). Lipid profiles were determined by F.P.L.C. (Dionex). HbA1c levels were determined from whole blood using the A1cNow monitoring kit (Metrika #0520105). For OGTT, mice were fasted for 16 h and then 2 g/kg body weight glucose in water was administered by oral gavage. BGL were determined at the indicated time points. For OLTT, mice were fasted for 16 h and then administered 8.35 µl/g olive oil by oral gavage. TGL levels were measured from whole blood using the CardioChek P.A. analyzer (PTS Inc.).

### Energy expenditure experiments

Energy expenditure, activity and respiratory quotient (RQ) were measured for 24 hours (dark and light phase) by using two open-circuit calorimetry systems (Integra system, AccuScan Instruments Inc., Columbus OH). Mice were allowed to adapt to the metabolic cages for 5 days. For measurements of oxygen consumption (VO_2_) and carbon dioxide production (VCO_2_), mice were placed in air-tight respiratory cages that were continuously ventilated with a flow rate of about 1 l/min. For each cage, air was sampled for 20 seconds at 2 min intervals. RQ was defined as VCO_2_ (L)/VO_2_ (L). RMR was calculated by taking the average of the 3 lowest VO_2_ readings in each experimental group during the light phase. Physical activity was determined by measuring beam breaks over a 24 h period. Physical activity was monitored by 3 arrays of 16 infrared light beam sensors and then converted into distance travelled in cm. Data were analyzed with AccuScan Integra ME software.

### Histopathology

Tissue samples were fixed in 4% paraformaldehyde, sectioned and stained with H&E according to standard methods. Histological appraisal was made by a qualified veterinary pathologist.

### Statistical analysis

For the analysis of body weight, blood glucose and fructosamine data two-way ANOVA were used. All other statistical analyses were made using a two-sided student's t-test. Areas under the curve for the OGTT were determined using Prism Graphpad software.

## Supporting Information

Figure S1 No cross reaction of Qβ-GIP induced antibodies with GLP1 and OXM *in vivo*. Qβ-GIP immunized mice. mice were challenged i.v. with 1 ng of I125-GIP, I125OXM or I125 GLP1. As a control naïve mice were challenged with 1 ng of I125-GIP. 30 minutes later the amount of antibody-bound GIP, OXM or GLP1 was determined. The percentage of antibody bound GIP, OXM or GLP-1±SEM (n = 4) is shown. Whereas most of the injected GIP was found associated with Antibodies only background levels of OXM or GLP1 were found associated with the antibody fraction in Qβ-GIP immunised mice.
(0.03 MB EPS)

Figure S2 Vaccination against GIP does not induce inflammation in the GIT. Female, C57BL/6 mice were immunized subcutaneously with 100 µg of Qβ-GIP or Qβ VLP on days 0, 14, 28 and 42. Animals were fed a high fat diet (35% fat w/v) from the start of the experiment. Mice were sacrificed on day 99. Tissue samples were fixed, sectioned and stained with H&E according to standard methods. One representative mouse from each group is shown. Histological analysis of the sample by a pathologist did not reveal any signs of inflammation in the gut in Qβ-GIP immunised animals compared to Qβ treated control animals. Similar results were obtained when animals were culled either on day 36 or 142 and analyzed by a pathologist.
(15.05 MB EPS)

Figure S3 Vaccination against GIP results in weight loss in obese male mice. Male mice were fed a high fat diet for 4 month. By this time all animals were severely obese and had reached weights between 45–50 g. The animals were then immunized (days 0, 14, 36, 50 and 119) with Qβ-GIP or Qβ VLPs and kept on a high fat diet (35% fat w/v). Fat content in the diet was reduced to 20% fat (w/v) from day 42 onwards. Average changes in body weight±SEM (n = 10) are shown. Qβ-GIP treated animals lost significantly more weight than Qβ VLP-immunized animals from day 70 onwards (two way ANOVA F(1,162) = 9.82, p = 0.0057).
(0.05 MB EPS)
